# Hypoxia increases expression of selective facilitative glucose transporters (GLUT) and 2-deoxy-d-glucose uptake in human adipocytes

**DOI:** 10.1016/j.bbrc.2007.07.032

**Published:** 2007-09-21

**Authors:** I. Stuart Wood, Bohan Wang, Silvia Lorente-Cebrián, Paul Trayhurn

**Affiliations:** Obesity Biology Unit, School of Clinical Sciences, University Clinical Departments, Royal Liverpool University Hospital, University of Liverpool, UK

**Keywords:** Hypoxia, HIF-1α, Human adipocytes, GLUT1, GLUT3, GLUT5, Adipokines, Obesity, Metabolic syndrome, Glucose transport, HIF-1α, hypoxia-inducible factor 1α, POLR2A, RNA II polypeptide A, GLUT, facilitative glucose transporter family, 2-DG, 2-deoxy-d-glucose

## Abstract

Hypoxia modulates the production of key inflammation-related adipokines and may underlie adipose tissue dysfunction in obesity. Here we have examined the effects of hypoxia on glucose transport by human adipocytes. Exposure of adipocytes to hypoxia (1% O_2_) for up to 24 h resulted in increases in GLUT-1 (9.2-fold), GLUT-3 (9.6-fold peak at 8 h), and GLUT-5 (8.9-fold) mRNA level compared to adipocytes in normoxia (21% O_2_). In contrast, there was no change in GLUT-4, GLUT-10 or GLUT-12 expression. The rise in GLUT-1 mRNA was accompanied by a substantial increase in GLUT-1 protein (10-fold), but there was no change in GLUT-5; GLUT-3 protein was not detected. Functional studies with [^3^H]2-deoxy-d-glucose showed that hypoxia led to a stimulation of glucose transport (4.4-fold) which was blocked by cytochalasin B. These results indicate that hypoxia increases monosaccharide uptake capacity in human adipocytes; this may contribute to adipose tissue dysregulation in obesity.

White adipose tissue is increasingly recognised as an important component of whole-body homeostasis. No longer regarded solely as energy storage cells, adipocytes secrete a large number of protein factors (adipokines) which are involved in a diverse range of biological functions, including energy balance, lipid metabolism, insulin sensitivity, angiogenesis, and haemostasis [1,2]. A large number of adipokines are related to inflammation and immunity, and these include TNFα, IL-1β, IL-6, IL-8, MCP-1, and MIF [2–4]. Obesity is characterised by a state of chronic, low-grade inflammation and white adipose tissue participates directly in this through the increased production of inflammation-related adipokines [2,5]. An exception is adiponectin, with its anti-inflammatory actions [6]. The recruitment of macrophages into adipose tissue is an important component of the inflammatory response during the development of obesity [7,8].

Inflammation in adipose tissue is increasingly considered to lead to the development of the disorders associated with obesity, such as type 2 diabetes and the metabolic syndrome [2,3,5,9]. However, the mechanistic basis for the inflammatory response as tissue mass expands is unknown. Suggestions include endoplasmic reticulum stress and oxidative stress [10,11]. We have proposed that hypoxia may act as a key trigger [2], and incubation of murine-derived adipocytes under low O_2_ tension leads to an induction in leptin, VEGF, visfatin, and PAI-1 expression [12–14]. In a study on human adipocytes we have now shown that the expression and secretion of several key pro-inflammatory adipokines are upregulated in hypoxic conditions induced either chemically or by low O_2_ tension [15]. These include IL-6, leptin, VEGF, angiopoietin-like protein 4, and MIF [15]. The expression and secretion of adiponectin, on the other hand, has been shown to fall in both human adipocytes and in 3T3-L1 cells [12,15,16]. Evidence for hypoxia occurring in adipose tissue *in vivo* in obesity has been presented for animal models [16].

The GLUT-1 facilitative glucose transporter gene and genes encoding glycolytic enzymes are recognised to be hypoxia-sensitive in many cells, expression being regulated through the hypoxia-inducible transcription factor, HIF-1 [17]. Increased GLUT-1 gene expression has been observed in human adipocytes in response to low O_2_ tension [15]. However, adipose tissue expresses several different GLUT isoforms [18,19] and in this study, we have investigated the effects of hypoxia on the expression of the different GLUT isoforms in human adipocytes. We show that GLUT-1, GLUT-3, and GLUT-5 gene expression (but not GLUT-4, GLUT-10, and GLUT-12) is increased by hypoxia, that GLUT-1 protein is also increased, and that these changes are accompanied by a hypoxia-induced increase in glucose transport by human adipocytes.

## Materials and methods

*Cell culture*. Cryopreserved human subcutaneous preadipocytes, derived from human adipose tissue of six female subjects (mean BMI 27.3; average age 39 years), were obtained (together with culture media) from Zen-Bio Inc. Cells were differentiated and cultured exactly as previously described [15]. Fully differentiated cells at day 14 post-induction were subjected to a hypoxic environment by placing in a MIC-101 modular incubator chamber (Billups-Rosenberg), which was flushed with 1% O_2_/94% N_2_/5% CO_2_, sealed and placed at 37 °C for up to 24 h as indicated. Control cells were cultured in a standard incubator (21% O_2_ and 5% CO_2_). All incubations at each time-point were performed in replicates of up to six wells. Human SGBS adipocytes (courtesy of M Wabitsch) were differentiated and cultured as previously described [18].

*Real-time PCR*. Total RNA was isolated directly from mature adipocytes (TRIZOL, Invitrogen), treated with DNase I (Invitrogen) and cDNA synthesised (Reverse-iT™ Kit, Abgene). Relative quantification of gene expression was measured by real-time PCR on a Mx3005P cycler (Stratagene) using the ^2−ΔΔ^*C*_t_ method [20]. All samples were normalized to values of POLR2A or β-actin and the results expressed as ‘fold change’ relative to controls. Primers were designed using Beacon Designer software (Premier Biosoft Int.) and the qPCR products detected using SYBR Green (Core kit, Eurogentec) incorporating a melt curve analysis for each run. Primer sequences are shown in Table 1.

*Immunoblot analysis*. Total protein lysates were prepared by collecting the cells in lysis buffer (0.5 M Tris, pH 6.8, 10% SDS, 10% glycerol, 1 mM PMSF, and 0.2 mM DTT) supplemented with Roche Complete proteinase inhibitor mix. Lysates were homogenised with a 23G syringe needle and the protein concentration determined using BCA reagent. Samples (30–40 μg/lane) were then separated by 10% SDS–PAGE and transferred to a nitrocellulose membrane (Hybond-ECL, GE Healthcare). Primary antibodies used were HIF-1α (R&D Systems), GLUT-1 (Prof S.A Baldwin, University of Leeds, UK), GLUT-5 (Dr S.W Cushman, NIH, USA) and α-tubulin (Sigma). Secondary antibodies, conjugated to HRP, were anti-rabbit (Serotech), anti-mouse (Santa Cruz), and anti-goat (R&D Systems). Signals were detected by enhanced chemiluminescence and developed using Hyperfilm-ECL (GE Healthcare). The membranes were successively placed in stripping buffer (62.5 mM Tris–HCl, pH 6.8, 2% SDS, and 100 mM β-mercaptoethanol) for 30 min at 50 °C, washed with PBS and subsequently reprobed. The intensity of the signals was quantified by scanning densitometry (Phoretix 1D Quantifier, Nonlinear Dynamics).

*Measurement of 2-deoxy-**d**-glucose transport*. Glucose transport into cells was determined using 2-deoxy-d-glucose (2-DG) based on a protocol from the Wabitsch laboratory (P. Fischer-Posovszky, personal communication). Cells cultured in 24-well plates were washed with PBS and then incubated in KRH buffer (130 mM NaCl, 10 mM Hepes, 10 mM MgSO_4_, 2.5 mM NaH_2_PO_4_, 4.6 mM KCl, and 2.5 mM CaCl_2_, pH 7.4) containing 1% BSA for 15 min at 37 °C, 5% CO_2_. 2-DG was added to a concentration of 60 μM containing 0.2 μCi/well of 2-deoxy-d-[1-^3^H]glucose (Sp. Act 315 GBq/mmol, GE Healthcare) for 5 min at 37 °C, 5% CO_2_. Uptake was stopped by the addition of 2 ml of ice-cold PBS containing 200 μM phloretin (Sigma). The cells were washed three times with PBS stop solution, solubilised in 0.1 N NaOH for 10 min at 22 °C. The cell lysates were subjected to liquid scintillation counting using EcoScint A fluid (National Diagnostics). For cells subjected to hypoxia, PBS and KRH buffers were stored in an atmosphere of 1% O_2_/94% N_2_/5% CO_2_ prior to addition to the cells. The uptake of 2-DG was measured in the absence and presence of 40 μM cytochalasin B to correct for non-specific uptake.

*Statistical analysis*. The results are expressed as mean values ± SE. Differences between groups were analysed by unpaired Student’s *t* tests.

## Results

### Expression of facilitative glucose transporter (GLUT) genes in hypoxia

**Fig. 1:**
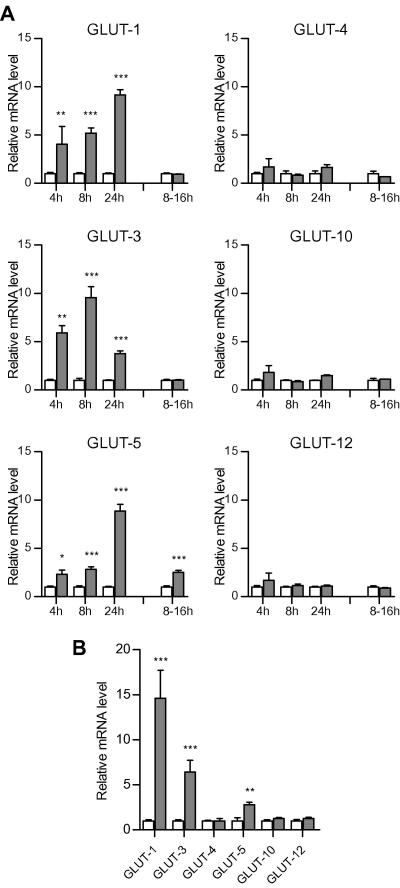
Facilitative glucose transporter gene expression in human adipocytes in hypoxia. Adipocytes at day 14 (post-induction of differentiation) were exposed to 21% or 1% O_2_ for up to 24 h. Total RNA was isolated and GLUT gene family mRNAs quantified by real-time PCR. Results are mean values ± SE (*n* = 4), expressed as relative to the control group. (A) ‘Zen-Bio’ adipocytes; (B) SGBS adipocytes. Twenty-one percent of O_2_ (open bars); 1% O_2_ (shaded bars). ^∗^*P* < 0.05; ^∗∗^*P* < 0.01; ^∗∗∗^*P* < 0.001, compared to adipocytes in normoxia.

Human adipocytes differentiated from preadipocytes (Zen-Bio) in culture were incubated in 21% or 1% O_2_ for 4, 8, and 24 h, and the levels of mRNA for specified GLUT gene family members assessed by real-time PCR. As shown in Fig. 1A, a significant increase (4-fold) was observed in the relative level of GLUT-1 mRNA by 4 h and at the subsequent time points. GLUT-1 mRNA level was highest at 24 h, with a 9.2-fold increase. When the cells were returned to 21% O_2_ (for 16 h) following exposure to 1% O_2_ for 8 h, GLUT-1 mRNA level returned to initial levels.

There was also a significant elevation in the level of GLUT-3 and GLUT-5 mRNAs in hypoxia at each of three time points examined (Fig. 1A). In the case of GLUT-3, the maximum increase was 9.6-fold at 8 h and the mRNA level returned to normal following 16 h recovery in normoxia. A significant increase in GLUT-5 mRNA was observed by 4 h at 1% O_2_ and the level increased to a maximum of 8.9-fold at 24 h. However, unlike GLUT-1 and GLUT-3, GLUT-5 mRNA remained unchanged following return of the cells to 21% O_2_ for 16 h (Fig. 1A). Analysis of GLUT-4, GLUT-10, and GLUT-12 revealed that in contrast to the previous GLUTs, there was no significant change in mRNA levels following exposure to low O_2_ tension (Fig. 1A).

To determine whether hypoxia-induced expression of GLUT-1, GLUT-3, and GLUT-5 is characteristic of human adipocytes, SGBS adipocytes were exposed to 1% O_2_ for 24 h. Similar findings to Zen-Bio adipocytes were observed in that increases in mRNA levels were found for GLUT-1 (14.6-fold), GLUT-3 (6.4-fold), and GLUT-5 (2.8-fold), whereas no significant change was detected for GLUT-4, GLUT-10 or GLUT-12 (Fig. 1B). One difference between the two adipocyte types was that while the increase in GLUT-5 mRNA in the Zen-Bio cells was higher than that of GLUT-3, this was opposite in the SGBS cell strain. The *C*_t_ values obtained under basal conditions for each of the GLUTs are shown in Table 1.

### Immunoblot analysis of GLUT proteins in hypoxia

**Fig. 2:**
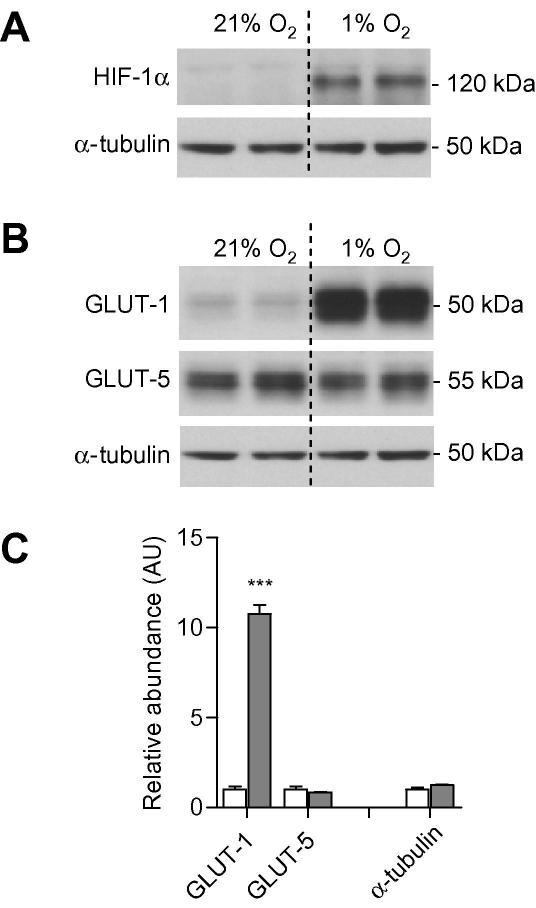
Facilitative glucose transporter protein expression in human adipocytes in hypoxia. Adipocytes at day 14 (post-induction of differentiation) were exposed to 21% or 1% O_2_ for 24 h. Total cellular lysates were isolated and western blot analysis performed for (A) HIF-1α, and (B) GLUT-1 and GLUT-5. Representative blots are shown. (C) Quantification of GLUT-1 and GLUT-5 proteins by densitometry normalised to α-tubulin. The densitometry values for each protein are set relative to the respective control as =1. *n* = 5 per group, AU = arbitrary units. Twenty-one percent of O_2_ (open bars); 1% O_2_ (shaded bars). ^∗∗∗^*P* < 0.001, compared to adipocytes in normoxia.

In the next experiments, the effect of hypoxia on GLUT protein levels was examined. Total cellular lysates prepared from the differentiated adipocytes were examined with antibodies to those GLUT family members which showed an increase in gene expression following exposure to 1% O_2_. Initially, induction of HIF-1α, the inducible subunit of the hypoxia-sensitive transcription factor HIF-1, was confirmed in the adipocytes by Western blotting (Fig 2A). The GLUT protein pattern is shown in Fig. 2B. When normalised to the α-tubulin signal, an increase of over 10-fold in GLUT-1 protein abundance was found in the hypoxic cells compared to the controls (Fig. 2C). However, there was no change in the abundance of GLUT-5. Despite repeated attempts with four different antibodies, we were unable to detect an unambiguous, specific signal for GLUT-3 protein.

### 2-Deoxy-d-glucose uptake in hypoxia

**Fig. 3:**
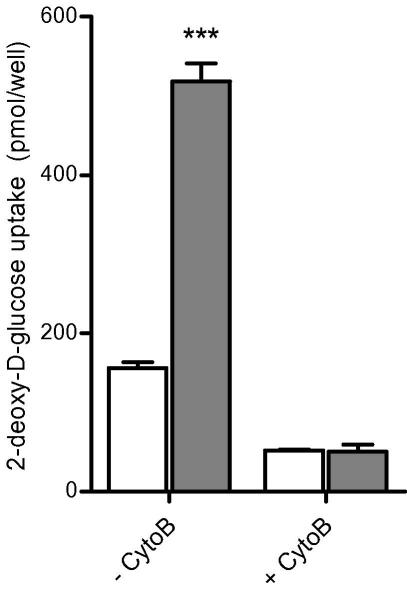
Uptake of 2-deoxy-d-glucose by human adipocytes in hypoxia. Adipocytes at day 14 (post-induction of differentiation) were exposed to 21% or 1% O_2_ for 24 h. Uptake of [^3^H]2-deoxy-d-glucose was measured in the absence and presence of 40 μM cytochalasin B (−/+ CytoB). The results are expressed as mean values ± SE, (*n* = 6, in three separate experiments). Twenty-one percent of O_2_ (open bars); 1% O_2_ (shaded bars). ^∗∗∗^*P* < 0.001, compared to adipocytes in normoxia.

To assess the functional consequences of increased GLUT expression following exposure of human adipocytes to 1% O_2_, the uptake of 2-DG, a non-metabolised analogue of d-glucose, was determined. The results in Fig. 3 show that 24 h exposure to 1% O_2_ led to a 3.3-fold increase in the uptake of 2-DG by the adipocytes. Incubation in the presence of the glucose transport inhibitor, cytochalasin B, resulted in a marked fall in 2-DG uptake with the complete abolition of the hypoxia-induced increase (Fig. 3). Correction of the uptake data for non-specific transport (uptake in the presence of cytochalasin B) indicates that hypoxia increased 2-DG uptake 4.5-fold.

## Discussion

We have proposed that hypoxia occurs in white adipose tissue as tissue mass increases during the development of obesity, and that this underlies the inflammatory response leading to obesity-associated diseases such as type 2 diabetes and the metabolic syndrome [2]. This concept is based on several observations, particularly that hypertrophied adipocytes are larger than the normal diffusion distance of O_2_ within tissues [14], that the proportion of the cardiac output to adipose tissue is not increased in the obese [21], and that obese subjects do not exhibit the post-prandial increase in blood flow to adipose tissue that occurs in the lean [22]. Several recent studies are consistent with this proposition, demonstrating increased expression and secretion of inflammation-related adipokines such as IL-6, leptin, MIF, and VEGF by adipocytes (including human) under hypoxic conditions [12–15]. In contrast, adiponectin production by adipocytes is inhibited by hypoxia [12,15,16].

There are multiple metabolic adaptations to a reduced O_2_ environment, with cells switching to anaerobic glycolysis thereby producing less cellular ATP per glucose molecule (Pasteur Effect). Consequently, the demand for glucose rises leading to an increase in the number of glucose transporters on the plasma membrane. The present study demonstrates that in human adipocytes exposure to hypoxia selectively regulates members of the GLUT transporter family. It also demonstrates that human adipocytes, like other cell types, increase their uptake of glucose in response to low O_2_ tension. The data presented would indicate that the induction of GLUT-1 is mainly responsible for the increased glucose uptake, both GLUT-1 mRNA and protein increasing markedly in cells maintained in hypoxic conditions. The increase in GLUT-1 mRNA is consistent with recent observation on human adipocytes [15] and on mouse 3T3-L1 cells [16]. Upregulation of GLUT-1 protein represents a response to chronic hypoxia [23], with GLUT-1 gene transcription being directly regulated by HIF-1α [24].

Increases in GLUT-3 and GLUT-5 gene expression were also observed in response to hypoxia in the present study. However, in the case of GLUT-3 we were unable to detect the protein itself—despite using different antibodies which provided a clear signal in tissues such as the brain in which this transporter is present. The apparent absence of GLUT-3 protein, or its presence at very low levels, would indicate that this GLUT isoform does not play a role in the hypoxia-induced stimulation in glucose transport by human adipocytes. Previous studies have also shown discordance between GLUT-3 mRNA levels and protein expression [25,26]. GLUT-3 gene expression has been observed previously in adipose tissue [27]. The signal observed here in cultured human adipocytes by real-time PCR occurred at a low *C*_t_ value (Table 1), indicating a relatively high abundance of the mRNA. We have also detected GLUT-3 mRNA by RT-PCR and confirmed its identity by DNA sequencing in both human adipocytes and 3T3-L1 cells (results not shown). GLUT-3 is not as widely characterised as GLUT-1, but it has recently been shown to be hypoxia-responsive in neural stem cells [28].

Although the expression of GLUT-5 was found to be upregulated during hypoxia, a corresponding increase in protein abundance was not detected following 24 h exposure to 1% O_2_. While its role as a fructose transporter means that GLUT-5 would not have contributed to the hypoxia-induced increase in glucose uptake, a requirement for fructose by hypoxic adipocytes has not been reported. Post-translational mechanisms may regulate the expression of the protein under hypoxic conditions in particular.

The absence of any response to hypoxia for GLUT-4, GLUT-10, and GLUT-12 gene expression suggests that these transporters do not contribute to the increased glucose uptake of hypoxic adipocytes. Similarly, the protein levels for GLUT-4 in total cell lysates remained unchanged (results not shown). Our results are similar to the finding that L6 muscle cells show no change in GLUT-4 protein levels in total plasma membranes in response to low O_2_ tension [29]. However, it has been reported that sequestered intracellular vesicles of GLUT-4 are translocated to the plasma membrane during acute hypoxia by a mechanism distinct from that occurring with insulin stimulation [30]. Acute translocation of GLUT-4 to the plasma membrane takes place independently of transcription or translation [31], and the possibility that such a process may occur in hypoxic adipocytes cannot be excluded. Similarly, GLUT-12 is thought to reside in intracellular vesicles and may be subject to translocation under parallel conditions [32]. However, no information is available with regard to a potential role in hypoxia for either GLUT-12 or GLUT-10.

The recruitment and activation of glycolytic enzymes by hypoxia are well established [33] and the increased influx of glucose would be expected to cause disruption to cellular glucose homeostasis. There is now considerable evidence in support of adipocytes being regulators of glucose homeostasis through both endocrine and non-endocrine mechanisms [34]. Indeed, the concept that the adipocyte can act as a glucose sensor has been proposed [35]. In this model, decreased glucose influx into the cell may provide signal cues that are released by the adipocyte. The situation in hypoxia where anaerobic glycolysis is enhanced may impose the opposite, but nonetheless detrimental, effect as a result of excess glucose influx.

In conclusion, prolonged exposure to hypoxia may lead to cellular dysfunction beyond that directly involving the production of adipokines, such as in disruption to cellular glucose and lipid metabolism; this may underscore the initiation and progression of obesity-related disorders.

## Figures and Tables

**Table 1:** Primer sequence data used for real-time PCR and mean *C*_t_ values obtained with adipocytes (Zen-Bio) under basal (normoxic) conditions

Gene	Sequence 5′–3′	Size (bp)	*C*_t_ value
GLUT-1	F: ATACTCATGACCATCGCGCTAG	93	25.7
	R: AAAGAAGGCCACAAAGCCAAAG		
			
GLUT-3	F: ACTTTGACGGACAAGGGAAATG	180	22.8
	R: ACCAGTGACAGCCAACAGG		
			
GLUT-4	F: TTCCAACAGATAGGCTCCGAAG	87	27.1
	R: AAGCACCGCAGAGAACACAG		
			
GLUT-5	F: GGAGCAACAGGATCAGAGC	89	21.4
	R: GGAAGGATGACCCAAAGGC		
			
GLUT-10	F: GCCTTCTGCAACAGCTTCAAC	82	25.1
	R: ACAAGCCGATGGTGCCAATG		
			
GLUT-12	F: TGCTTGTTTATGTTGCTGCTTTTT	86	28.8
	R: TGATCCCACCAGGAAAGATCTC		
			
POLR2A	F: ATGGAGATCCCCACCAATATCC	81	26.2
	R: CATGGGACTGGGTGCTGAAC		
			
β-Actin	F: TTGCCGACAGGATGCAGAA	101	n.a
	R: GCCGATCCACACGGAGTACT		

n.a., not applicable.
